# Identification of *Brassica oleracea *proteins during early infection by *Xanthomonas campestris pv.campestris*

**DOI:** 10.1186/1753-6561-8-S4-P97

**Published:** 2014-10-01

**Authors:** Juan Costa, Gabriela Villeth, Luciano Paulino, Mateus Santos, Angela Mehta

**Affiliations:** 1 Universidade de Brasília - Campus Darcy Ribeiro, Brasília, DF, 70910-900, Brazil; 2 Embrapa Recursos Genéticos e Biotecnologia, Av W5 Norte Final, 70770-910, Brasilia, DF, Brazil; 3 Embrapa Hortaliças, Brasília, DF, Brazil

## Background

The family Brassicaceae comprises several important crops cultivated in Brazil, including broccoli, cauliflower and cabbage. One of the main diseases that affects all cruciferous plants is black rot, caused by the bacterium *Xanthomonas campestris *pv. *campestris *(Xcc). This bacterium causes serious damage to the plant, leading to severe yield losses. The disease control is extremely difficult since the seeds are the main source of bacterial dissemination. The aim of this work was to identify proteins from *Brassica oleracea *during early infection by Xcc, in an attempt to identify proteins related to resistance.

## Methods

Plants from the resistant (União) and susceptible (Kenzan) cabbage genotypes were inoculated with the bacterium and leaves were collected at 24 hours after inoculation (hai). Approximately 0.1 g of tissue was used for protein extraction using phenol. The proteins were quantified and approximately 600 μg of total protein was subjected to two-dimensional electrophoresis (2-DE). The analysis of the 2D maps were performed with the ImageMaster 2D Platinum v7.0 software (GE Healthcare) using three gels from each condition.

## Results and conclusions

A comparison between the 2D maps of the inoculated plants and the control condition of the resistant and susceptible genotypes was performed. The analysis of the resistant genotype revealed 22 differential proteins, including 2 exclusive to Xcc inoculated plants and 2 to the control condition. In the resistant interaction, most proteins showed decreased intensity in response to Xcc. On the other hand, in the susceptible interaction, most differential proteins were increased upon Xcc infection. One of the proteins identified was a peroxiredoxin precursor, which was decreased in the susceptible genotype inoculated with Xcc. Proteins involved in photosynthesis were also modulated by Xcc infection. The results obtained may help better understand the susceptible and resistant interactions of *B. oleracea*-Xcc.

